# A Nanoparticle-Based Strategy to Stabilize 5-Azacytidine and Preserve DNA Demethylation Activity in Human Cardiac Fibroblasts

**DOI:** 10.3390/pharmaceutics18010088

**Published:** 2026-01-09

**Authors:** Kantaporn Kheawfu, Chuda Chittasupho, Sudarshan Singh, Siriporn Okonogi, Narainrit Karuna

**Affiliations:** 1 Department of Pharmaceutical Science, Faculty of Pharmacy, Chiang Mai University, Chiang Mai 50200, Thailand; kantaporn.kheawfu@cmu.ac.th (K.K.);; 2 Center of Excellence in Pharmaceutical Nanotechnology, Faculty of Pharmacy, Chiang Mai University, Chiang Mai 50200, Thailand; 3 Office of Research Administration, Chiang Mai University, Chiang Mai 50200, Thailand; 4 Department of Pharmaceutical Care, Faculty of Pharmacy, Chiang Mai University, Chiang Mai 50200, Thailand

**Keywords:** 5-Azacytidine, DNA methylation, lipid nanoparticle, drug stability, epigenetic therapy

## Abstract

**Background:** 5-Azacytidine (5-Aza) is a clinically important DNMT inhibitor with the potential to modulate cardiac remodeling by epigenetically reprogramming human cardiac fibroblasts (HCFs). However, its clinical utility is limited by rapid hydrolytic degradation. Nanoparticle (NP) encapsulation offers a strategy to mitigate this instability. This study evaluated the physical and chemical stability of free 5-Aza and 5-Aza-loaded lipid nanoparticles (5-Aza-NP) under different storage temperatures and examined their effects on DNA methylation-related gene expression in HCFs. **Methods:** Hyaluronic acid-stabilized lipid NPs were prepared using a solvent displacement method. Particle size, polydispersity index (PDI), and zeta potential were monitored over four days at −20 °C, 4 °C, and 30 °C. Chemical stability was assessed using HPLC and first-order kinetic modeling. Functional activity was evaluated by treating HCFs with free 5-Aza or 5-Aza-NP stored for 96 h and measuring *DNMT1*, *DNMT3A*, and *DNMT3B* expression by RT-qPCR. **Results:** 5-Aza-NP remained physically stable at 4 °C, while −20 °C induced aggregation and 30 °C caused thermal variability. Free 5-Aza degraded rapidly at 30 °C (6.56% remaining at 72 h), whereas 5-Aza-NP preserved 11.54%. Kinetic modeling confirmed first-order degradation, with consistently longer half-lives for the NP formulation. Functionally, 5-Aza–NP preserved its ability to suppress *DNMT1* expression following 96 h of storage at 4 °C, whereas free 5-Aza showed reduced activity. In contrast, *DNMT3A* and *DNMT3B* levels remained low and unchanged across all treatments. **Conclusions:** NP encapsulation enhances the physicochemical stability of 5-Aza and preserves its *DNMT1*-inhibitory activity, while *DNMT3A/B* remain unaffected. These findings support NP-based delivery as a promising strategy to stabilize labile epigenetic drugs such as 5-Aza.

## 1. Introduction

In recent years, epigenetic alterations and cardiovascular diseases have been in the spotlight [1]. Epigenetics refers to heritable changes in gene expression that occur without altering the underlying DNA sequence. One key mechanism is DNA methylation, in which DNMT1, DNMT3A, and DNMT3B catalyze the addition of a methyl group to the 5-carbon of cytosine, generating 5-methylcytosine [2]. DNA methylation contributes to cardiac remodeling, and treatment with 5-Azacytidine (5-Aza) has shown beneficial effects, supporting its potential as a therapeutic epigenetic modulator in cardiac hypertrophy and fibrosis [3,4]. 5-Aza is a cytidine analog widely used in the treatment of hematologic malignancies such as myelodysplastic syndromes and acute myeloid leukemia [5]. Its therapeutic effect is attributed to incorporation into nucleic acids and subsequent inhibition of DNA methyltransferase, leading to hypomethylation and reactivation of silenced tumor suppressor genes [6]. Despite its established clinical relevance, the broader application of 5-Aza remains severely constrained by its pronounced chemical instability [7]. In aqueous solution, 5-Aza undergoes rapid hydrolytic degradation through ring opening and cleavage of the triazine moiety, producing inactive by-products that reduce its therapeutic efficacy [8]. Exposure to elevated temperatures and neutral or alkaline pH substantially accelerates its degradation, resulting in a short half-life and restricted shelf-life under routine storage conditions [9]. The chemical stability of 5-Aza has been previously evaluated. Walker et al. reported that 5-Aza suspensions at 25 mg/mL showed temperature-dependent stability. With 97.5% confidence, >90% of the initial 5-Aza concentration will remain intact if total storage time is limited to ≤2 h at 23 °C, ≤8 h at 4 °C, and ≤4 days at −20 °C. This can cause drug expenditures [7]. Additionally, 5-Aza at 0.2 mg/mL and 2.0 mg/mL in glass or plastic i.v. bottle bags showed rapid degradation, with all t_90_ values (time at which 90% of the initial concentration remained) <3 h. At 2.0 mg/mL, t_90_ values ranging from 2.4–3.0 h indicated short-life stability [10]. These results underscore the need for an innovative approach to 5-Aza storage that maintains clinically relevant effects.

To address these challenges, formulation strategies have been explored to enhance the stability and therapeutic potential of 5-Aza. Thus, nanoparticle (NP)-based drug delivery systems are particularly attractive due to their ability to protect labile drugs from hydrolytic degradation, improve pharmacokinetics, and enable targeted delivery. Cheng et al. demonstrated that FITC-labeled mesoporous silica nanoparticles (NPs) can efficiently deliver 5-Aza into P19 embryonic carcinoma stem cells and enhance antiproliferative effects, upregulate cardiac differentiation markers, and induce histone modifications [11]. In another study, solid lipid NPs encapsulating 5-Aza demonstrated greater cytotoxicity against the MCF-7 cell line than the free drug, with apoptotic nuclear changes and time-dependent NP uptake observed [12]. Altogether, encapsulation of 5-Aza into NPs may provide a protective microenvironment that minimizes direct exposure to water and temperature fluctuations, thereby prolonging its stability during storage and administration.

**Figure 1:**
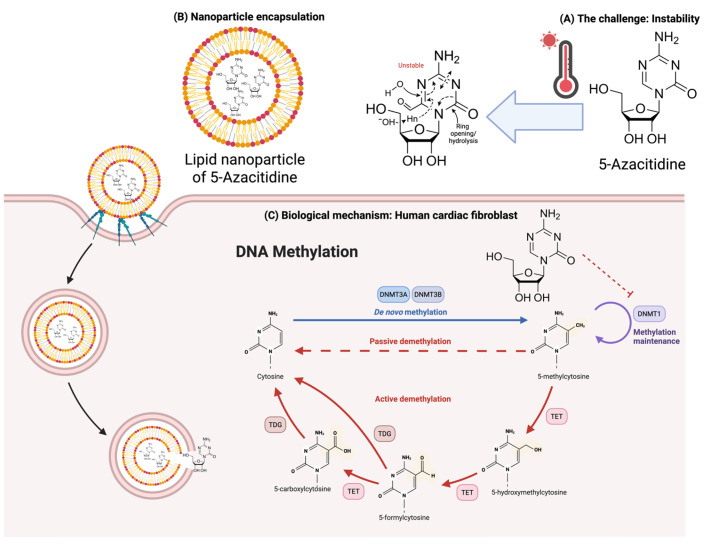
Schematic illustration of the proposed 5-Aza-NP stabilization and mechanism of action. (**A**) Free 5-Azacytidine (5-Aza) is susceptible to rapid hydrolytic degradation, especially at elevated temperatures, resulting in inactive by-products. (**B**) Encapsulation of 5-Aza into lipid nanoparticles stabilized with Hyaluronic Acid (HA) protects the drug from the aqueous environment, enhancing physicochemical stability. (**C**) Upon uptake by human cardiac fibroblasts, the stabilized 5-Aza-NP releases its payload, which enters the nucleus and inhibits DNA Methyltransferase 1 (*DNMT1*). This preservation of epigenetic activity allows for effective DNA demethylation, whereas the free drug loses efficacy due to degradation. DNMT1 = DNA Methyltransferase 1; DNMT3A/B = DNA Methyltransferase 3 A/B; TET = Ten-eleven Translocation and TDG = Thymine DNA Glycosylase. Created in BioRender. CMU, P. (2026) https://BioRender.com/clqimfl.

In this study, lipid NP formulations of 5-Aza, stabilized and targeted with hyaluronic acid (HA), were prepared using a solvent displacement method. The physical and chemical stability of the formulations was evaluated under different storage conditions. To establish whether physicochemical stabilization translates into preservation of epigenetic function, the ability of freshly prepared and stored 5-Aza and 5-Aza-loaded NPs to modulate *DNMT1*, *DNMT3A*, and *DNMT3B* expression in TGF-β1-stimulated human cardiac fibroblasts was further examined. By comparing both the stability profiles and *DNMT* expression responses between free 5-Aza and NP-encapsulated formulations, this work aims to clarify the extent to which NP encapsulation alleviates 5-Aza’s inherent chemical lability while preserving its DNA demethylation activity in cardiac fibroblasts. Indeed, the findings will contribute to the development of more stable and biologically effective delivery systems for this clinically significant epigenetic drug (Figure 1).

## 2. Materials and Methods

DNA methyltransferase (DNMT) inhibitor, 5-Azacitidine (5-Aza; A2385) was purchased from Sigma-Aldrich, Gillingham, UK. Acetonitrile (HPLC Grade), methanol (HPLC Grade), ammonium acetate (AR Grade), DMSO (AR Grade), and acetic acid (AR Grade) were purchased from RCI Labscan Limited, Bangkok, Thailand. Ortho-phosphoric acid (AR Grade) was purchased from Merck, Darmstadt, Germany. All other reagents in this study were obtained from certified sources.

### 2.1. Preparation of 5-Aza-Nanoparticle (5-Aza-NP)

5-Aza encapsulated nanoparticle (5-Aza-NP) formulations were prepared by solvent displacement method. Hyaluronic acid (HA) was first dissolved in phosphate-buffered saline (PBS) to obtain a 0.1% (*w*/*v*) solution, while a phosphatidyl choline stock suspension was prepared at a concentration of 40 mg in 4 mL of absolute ethanol. In parallel, 5-Aza was dissolved at 10 mg in 1 mL of dimethyl sulfoxide (DMSO) to generate a concentrated stock solution. Blank NP–HA prepared by infusing 1 mL of phosphatidyl choline solution into 10 mL of 0.1% HA in PBS. The 5-Aza-NP was prepared by mixing 1 mL of phosphatidyl choline solution with 250 µg of 5-Aza and infusing into 10 mL with 0.1% HA in PBS (at an injection rate of 3 mL/h); 5-Aza in PBS was prepared by dissolving 250 µg of 5-Aza in 10 mL PBS containing 1 mL ethanol.

### 2.2. Characterization of 5-Aza-NP

To assess characteristics of 5-Aza-NP, the mean particle size, polydispersity index (PDI), and zeta potential of the 5-Aza-NP (1.3 mg/mL) were determined by dynamic light scattering (DLS) at a scattering angle of 173 °C and 25 °C using a Zetasizer Nano Series (Malvern Instruments, Malvern, UK).

The morphology of the 5-Aza-NP was analyzed using transmission electron microscopy (TEM) (Hitachi HT7700, Hitachinaka, Ibaraki, Japan). A Formvar-coated copper grid (300 mesh, Sigma-Aldrich, St. Louis, MO, USA) was immersed in the nanoparticle suspension for 1 min to facilitate particle adsorption onto the grid surface. The excess solution was carefully removed, and the grid was subsequently stained with 2% (*w*/*v*) uranyl acetate for 30 s to enhance contrast. The grid was then air-dried in a desiccator to eliminate moisture. TEM imaging was conducted at 75 kV following the drying process, and NPs were captured at a magnification of 50,000×.

### 2.3. Determination of the Physical Stability of 5-Aza

The physical stability of 5-Aza-NP was assessed under different storage conditions. NP suspensions in deionized water were sealed in airtight containers and stored at −20 °C, 4 °C, and 30 °C for up to 4 days (96 h). At predetermined time points (0, 1, 2, 3, and 4 days), particle size, PDI, and zeta potential were measured by dynamic light scattering (Zetasizer ZS, Malvern Instruments, Malvern, UK) to evaluate the effect of storage conditions on NP stability.

### 2.4. Quantification of 5-Aza-NP Formulations

Encapsulation efficiency and drug loading of 55-Aza-NPs were quantified by HPLC analysis following nanoparticle separation. The 5-Aza-NP was prepared in DMSO:water (1:1 *v*/*v*), then centrifuged at 12,000 rpm for 10 min (4 °C) to separate the NPs. The supernatant was collected, filtered through a 0.45 µm nylon membrane, and analyzed by HPLC. HPLC analysis was performed using an Agilent 1260 Series system (Agilent Technologies, Pittsburgh, PA, USA) equipped with a quaternary pump, autosampler, column oven, and diode array detector. Reversed-phase HPLC separation was achieved on a C18 column (Thermo Fisher Scientific, Waltham, MA, USA; 250 × 4.6 mm, 5 µm). The chromatographic strategy was adapted from Marineni and Reddy (2014) [13]. Mobile phase A was 3.1 g ammonium acetate in 1000 mL water (pH 6.4 ± 0.05, adjusted with diluted acetic acid), and mobile phase B was a mixture of mobile phase A:methanol:acetonitrile (50:30:20 *v*/*v*). The gradient program included Time/% mobile phase B is 0 min/0%, 15 min/0%, 30 min/20%, 45 min/40%, 55 min/50%, 60 min/0%. The flow rate was set at 1.0 mL/min, column temperature at 35 °C, detection wavelength at 242 nm, and injection volume at 20 µL. A standard curve of 5-Aza (10–200 µg/mL) was also prepared in DMSO:water (1:1 *v*/*v*). Data were processed using Agilent ChemStation software (version C.01.08).

### 2.5. Stability Assessment of Free 5-Aza and 5-Aza-NP

The chemical stability of free 5-Aza and 5-Aza-NP was evaluated under controlled storage conditions. Aliquots of each formulation were stored at −20 °C, 4 °C, and 30 °C and sampled at predetermined time points (0, 1, 2, 3, and 4 days). At each time point, samples were retrieved, equilibrated to room temperature, and processed immediately to prevent further degradation. Suspensions were passed through a 0.45 μm membrane filter to remove particulates, and the filtrates were analyzed by HPLC. Peak areas corresponding to intact 5-Aza were integrated and expressed as percentage of drug remaining relative to Day 0.

In line with standard pharmaceutical stability conditions, we selected storage temperatures, including −20 °C, 4 °C, and 30 °C to assess stability of 5-Aza. Specifically, −20 °C reflects standard frozen preservation used to stabilize NPs and other labile drugs, and prior studies have shown minimal 5-Aza degradation over 23 days under these conditions [7]. Additionally, 4 °C reflects routine refrigerated handling conditions used in clinical practice, where 5-Aza degradation proceeds more slowly than at ambient temperature, while 30 °C serves as an accelerated stress condition aligned with ICH Q1 for the stability testing conditions for Climatic Zone IV regions.

To characterize degradation behavior, concentration–time data were fitted to multiple kinetic models, including zero-order, first-order, Higuchi, and Korsmeyer–Peppas models. Linear regression was applied to each model, and goodness-of-fit was assessed using the coefficient of determination (R^2^) to identify the predominant kinetic mechanism. For the best-fit model, the degradation rate constant (k) was extracted from the slope of the linearized plot, and the corresponding half-life (t½) and shelf-life (t90, time to 90% remaining) were calculated using standard kinetic equations.

### 2.6. Cell Culture Using Human Cardiac Fibroblasts (HCFs)

Human cardiac fibroblasts (HCFs) were used in the present study, as they exhibit inducible *DNMT* expressions upon TGF-β1 stimulation, a potent inducer of fibroblast differentiation [14]. HCFs were grown in Dulbecco’s Modified Eagle Medium (DMEM; G4511, ServiceBio, Wuhan, China) supplemented with 10% fetal bovine serum (FBS; F7524, Sigma-Aldrich, St. Louis, MO, USA) and 1% Penicillin–Streptomycin–Amphotericin B solution (G4015, ServiceBio, Wuhan, China). Cultures were maintained at 37 °C in a humidified atmosphere containing 5% CO_2_. When cells reached approximately 70–80% confluence, they were detached using 0.25% trypsin–EDTA (SH3004202, Hyclone, Logan, UT, USA) and subcultured. All assays were conducted with cells between passages 5 and 8 to ensure reproducibility and minimize variability.

### 2.7. Treatment of HCFs with 5-Aza and 5-Aza-NP Under Different Conditions

HCFs were stimulated using TGF-β1 (2 ng/mL) for 24 h to enhance *DNMTs* expression. Then, the effect of storage temperature and duration on the biological activity of 5-Aza (5 µM) and its nanoparticle formulation (5-Aza-NP, equivalent to 5 µM 5-Aza) were then evaluated by assessing their ability to modulate *DNMTs* in TGF-β1–treated HCFs. Aliquots of free 5-Aza, Blank NP, and 5-Aza-NP were stored at −20 °C, 4 °C, or 30 °C for 0, 1, 2, 3, and 4 days. At each designated time point, a stored aliquot was removed, equilibrated to room temperature, and used immediately for cell treatment.

HCFs were seeded into 6-well plates at a density of 6 × 10^3^ cells per well and allowed to adhere for 24 h. Cells were then assigned to the following groups: untreated control, free 5-Aza stored at −20 °C, 4 °C, or 30 °C (for 4 days), and 5-Aza-NP stored at −20 °C, 4 °C, or 30 °C (for 4 days). A constant drug concentration was applied across all 5-Aza–containing treatments. Blank NP was administered at the same NP concentration used for the 5-Aza-NP group. The treatments were maintained for 24 h at 37 °C in a humidified incubator containing 5% CO_2_. Following treatment, cells were harvested by trypsinisation, pelleted by centrifugation at 1000× *g* for 5 min, and stored at −80 °C until analysis.

### 2.8. Quantification of DNA Methylation–Related Gene Expression by RT-qPCR

Total RNA was extracted from treated HCF cells using the RNeasy Mini Kit (Qiagen, Hilden, Germany) according to the manufacturer’s instructions. Cell pellets were lysed in RLT buffer supplemented with 1% β-mercaptoethanol to ensure complete disruption and RNase inactivation. The lysates were homogenized by pipetting, mixed with 70% ethanol, and transferred onto silica-based spin columns to allow RNA binding. After washing with RW1 and RPE buffers, RNA was eluted in RNase-free water and stored at −80 °C. RNA concentration and purity were assessed using a NanoDrop™ (Thermo Fisher Scientific, Waltham, MA, USA), and samples with A_260_/A_280_ ratios between 1.8 and 2.1 were considered acceptable.

For cDNA synthesis, 1 µg of total RNA was treated with DNase I to remove genomic DNA contamination and subsequently reverse-transcribed using the High-Capacity cDNA Reverse Transcription Kit (Applied Biosystems, Waltham, MA, USA). Quantitative PCR was performed using LightCycler^®^ SYBR Green I Master Mix (Roche, Basel, Switzerland) on a QuantStudio™ 6 Real-Time PCR System (Applied Biosystems, Waltham, MA, USA). All reactions were prepared in triplicate. β-2-Microglobulin (*B2M*) was used as the internal reference gene, and relative gene expression levels were calculated using the 2^−ΔΔCt^ method. Primer sequences (5′→3′) were as follows: *B2M*: forward GATGAGTATGCCTGCCGTGT, reverse TGCGGCATCTTCAAACCTCC; *DNMT1*: forward GGCGGCTCAAAGATTTGGAA, reverse CAGGTAGCCCTCCTCGGATA; *DNMT3A*: forward GGCCATACGGTGGAGCC, reverse TGTTGAGCCCTCTGGTGAAC; *DNMT3B*: forward TCCCTGGCGGTCGGG, reverse TCCCTTCATGCTTTCCTGCC.

### 2.9. Statistical Analysis

All data were expressed as mean  ±  SD unless otherwise specified. The normality of data distribution was assessed using the Shapiro–Wilk test. Statistical analyses were carried out using GraphPad Prism 10 (GraphPad Software, San Diego, CA, USA). For comparisons involving more than three groups, one-way ANOVA followed by Tukey’s post hoc test was applied to normally distributed data, whereas the Kruskal–Wallis test with appropriate post hoc analysis was used for non-parametric datasets. A *p*-value < 0.05 was considered to indicate statistical significance. All experiments were performed in triplicate and repeated at least three times independently.

## 3. Results

### 3.1. Colloidal Characteristics of 5-Aza-NP

**Figure 2:**
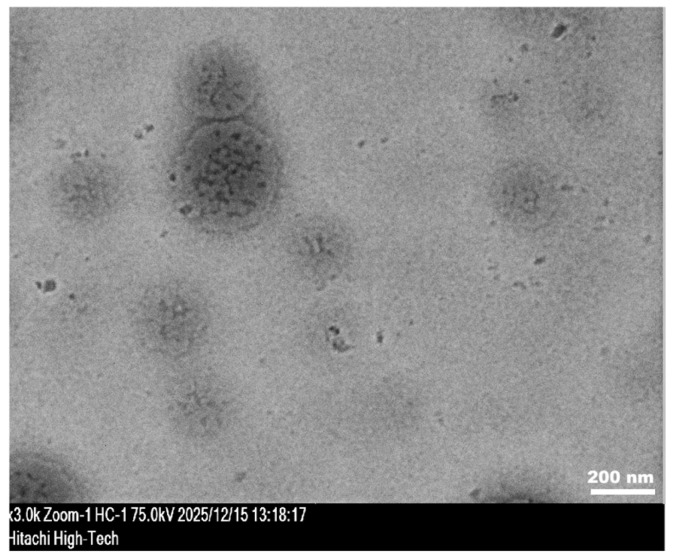
Transmission electron microscopy (TEM) image of 5-Aza-NPs.

Transmission electron microscopy (TEM) was employed to examine the morphology of 5-Aza-NPs. As shown in Figure 2, the NPs exhibited a predominantly spherical morphology with nanoscale dimensions. The particles appeared as discrete entities with no evidence of large-scale aggregation, indicating successful nanoparticle formation.

**Figure 3:**
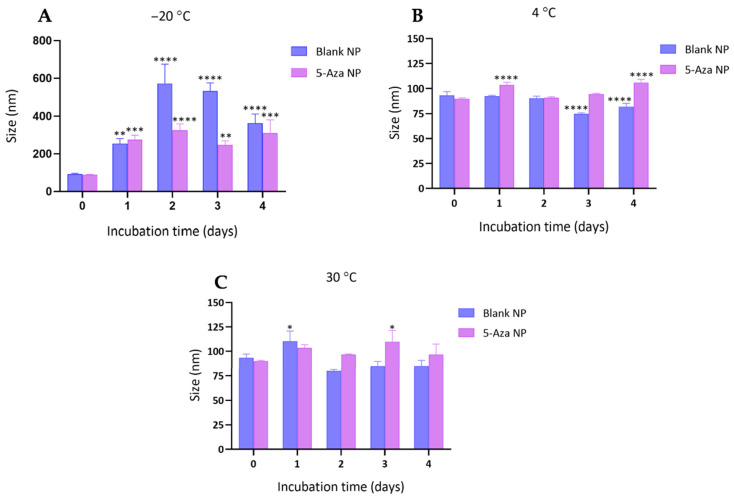
Effect of storage temperature on the particle size of blank nanoparticles (blank NP) and 5-Aza-loaded nanoparticles (5-Aza-NP) at (**A**) −20 °C (**B**) 4 °C and (**C**) 30 °C over time. Data are presented as mean ± SD (n = 3). Asterisks indicate significant differences compared with the corresponding Day 0 value: * *p* < 0.05, ** *p* < 0.01, *** *p* < 0.001, **** *p* < 0.0001.

The particle size of blank-NPs and 5-Aza-NP was monitored over four days under different storage conditions (−20 °C, 4 °C, and 30 °C) (Figure 1). At −20 °C, both formulations exhibited a marked increase in particle size compared to their initial measurements. Blank NPs increased from 93.3 ± 3.9 nm (Day 0) to 362.8 ± 48.8 nm (Day 4). In contrast, 5-Aza-NP showed relatively smaller size growth, increasing from 89.9 ± 0.8 nm (Day 0) to 310.4 ± 70.5 nm (Day 4) (Figure 3A). These results suggest that while both formulations aggregated at −20 °C, the extent of size enlargement was less pronounced in the drug-loaded system. Regarding storage at 4 °C, particle size remained relatively stable across the 4 days. Blank NPs showed a decrease in size to 82.1 ± 3.2 nm (Day 4), while 5-Aza-NP was slightly larger, with a value of 105.8 ± 3.2 nm (Day 4) (Figure 3B). These results indicate that refrigerated storage (4 °C) maintained colloidal stability, with only minor variations over time. At 30 °C, the size of Blank NP decreased to 85.0 ± 5.8 nm (Day 4). Aza NPs were stabilized at 96.7 ± 10.8 nm through Day 4 (Figure 3C).

**Figure 4:**
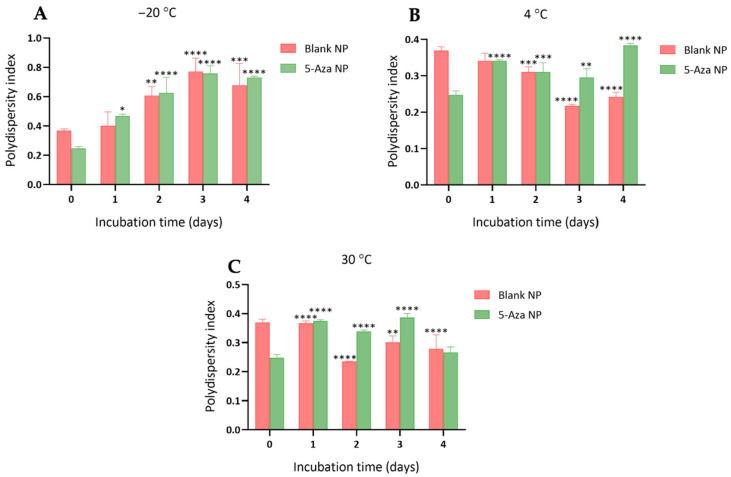
Effect of storage temperature on the polydispersity index (PDI) of blank nanoparticles (blank NP) and 5-Aza-loaded nanoparticles (5-Aza-NP) at (**A**) −20 °C (**B**) 4 °C and (**C**) 30 °C over time. Data are presented as mean ± SD (n = 3). Asterisks indicate significant differences compared with the corresponding Day 0 value: * *p* < 0.05, ** *p* < 0.01, *** *p* < 0.001, **** *p* < 0.0001.

The PDI values of blank NPs and 5-Aza-NP were evaluated to assess colloidal uniformity during storage at −20 °C, 4 °C, and 30 °C (Figure 4). At −20 °C, PDI values increased substantially over time, indicating particle aggregation and loss of homogeneity. Blank NPs showed a progressive increase from 0.37 ± 0.01 at Day 0 to 0.678 ± 0.149 at Day 4. Similarly, 5-Aza-NP increased from 0.248 ± 0.011 at Day 0 to 0.730 ± 0.009 by Day 4 (Figure 4A). This suggests that storage at −20 °C compromised colloidal stability in both formulations. At 4 °C, PDI values remained consistently low, indicating greater colloidal stability and a narrower particle size distribution. For Blank NPs, PDI values ranged from 0.218 ± 0.004 to 0.341 ± 0.021, while 5-Aza-NP showed values between 0.296 ± 0.024 and 0.384 ± 0.005 over the four-day storage period (Figure 4B). These results demonstrate that refrigerated conditions effectively preserved uniformity for both NP formulations. Furthermore, PDI values showed greater variation but remained within a moderate range during 30 °C storage (Figure 4C). Blank NPs showed the PDI value of 0.279 ± 0.048 on Day 4, while 5-Aza-NP remained relatively stable, showing the PDI value of 0.266 ± 0.019 on Day 4.

In addition, number-based DLS size-distribution profiles (Figure S1) show a narrow, unimodal particle population predominantly below 200 nm, confirming formulation homogeneity. The absence of secondary peaks indicates minimal aggregation and good colloidal stability. As number-weighted distributions reduce bias from rare large particles that can skew intensity-weighted means, these data provide a more accurate representation of the true particle population and support the TEM morphological observations.

**Figure 5:**
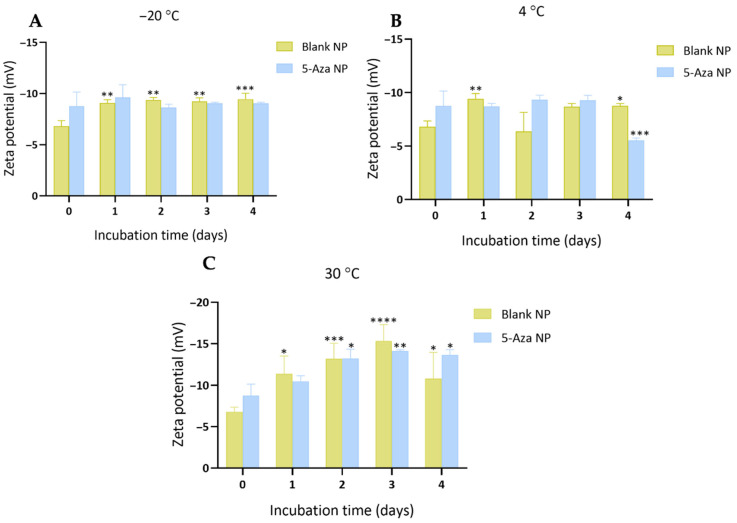
Zeta potential changes of blank nanoparticles (Blank NP) and 5-Aza-loaded nanoparticles (5-Aza NP) during storage at (**A**) −20 °C (**B**) 4 °C and (**C**) 30 °C over time. Data are presented as mean ± SD (n = 3). Asterisks indicate significant differences compared with the corresponding Day 0 value: * *p* < 0.05, ** *p* < 0.01, *** *p* < 0.001, **** *p* < 0.0001.

The surface charge of blank NPs and 5-Aza-NP was evaluated by measuring zeta potential (ZP) at different storage temperatures (Figure 5). At −20 °C, both formulations exhibited moderately negative zeta potential values. Blank NPs ranged from −6.8 ± 0.55 mV at Day 0 to −9.5 ± 0.58 mV at Day 4, while 5-Aza-NP showed a zeta potential value of −9.04 ± 0.12 mV (Figure 5A). Both remained moderately negative, with 5-Aza-NP slightly more negative than Blank NPs. At 4 °C, blank NPs varied from −9.4 ± 0.48 mV at Day 1 to −6.4 ± 1.78 mV at Day 2, before stabilizing around −8.8 ± 0.21 mV at Day 4. 5-Aza-NP were generally more negative, ranging from −9.3 ± 0.42 mV to −9.3 ± 0.45 mV during the first three days, but decreased to −5.5 ± 0.23 mV at Day 4 (Figure 5B). This reduction in negativity suggests a change in surface charge for the 5-Aza-NP formulation at 4 °C. At 30 °C, blank NPs exhibited a value of −10.82 ± 3.12 mV at Day 4, while 5-Aza-NP showed a value of −13.63 ± 0.65 mV (Figure 5C). These strongly negative charges indicate that, despite the elevated temperature, both formulations retained sufficient electrostatic repulsion to prevent immediate aggregation.

### 3.2. Encapsulation Efficiency, Drug Loading, and Chemical Stability of Free 5-Aza in PBS and NPs

**Figure 6:**
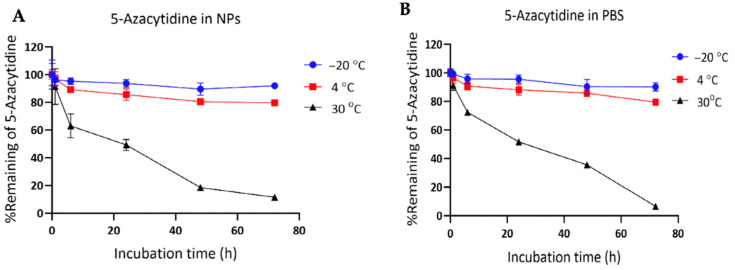
Chemical stability of (**A**) 5-Aza in phosphate-buffered saline (PBS) and (**B**) in 5-Aza-NPs under different storage temperatures.

The representative HPLC chromatograms (Figure S2) show a well-defined 5-Aza peak in the standard and PBS samples, while a corresponding peak is clearly observed in the 5-Aza-NPs, confirming successful drug incorporation. Quantitative analysis revealed an encapsulation efficiency of 83.92 ± 7.70% and a drug loading of 2.78 ± 0.26%, demonstrating efficient incorporation of 5-Aza into the nanoparticle matrix and supporting the suitability of this formulation for drug delivery applications. In Figure 6, both 5-Aza-NP and free 5-Aza remained relatively stable over 72 h at –20 °C. The free 5-Aza drug showed similar stability, i.e., 90.11 ± 2.90% at 72 h (Figure 4A). For 5-Aza-NP, the remaining percentage was 91.91 ± 1.80% at 72 h (Figure 4B). At 4 °C, both formulations, free 5-Aza and 5-Aza-NP, displayed gradual degradation. The 5-Aza-NP retained 79.67 ± 1.90% at 72 h, while the free 5-Aza drug decreased to 79.46 ± 0.42% at 72 h. Both formulations exhibited comparable stability, though NPs maintained slightly higher levels at early time points (Figure 6A,B). Interestingly, substantial differences were observed at 30 °C. The 5-Aza-NP formulation maintained 11.54 ± 0.64% of the initial drug content at 72 h, whereas the free 5-Aza drug degraded markedly faster, with only 6.56 ± 0.25% remaining at the same time point. Although both forms were unstable at 30 °C, the 5-Aza-NP provided enhanced protection, retaining nearly two-fold more drug than the free 5-Aza form at 72 h (11.54% vs. 6.56%), *p* < 0.01 (Figure 6A,B). Overall, these findings underline that both the 5-Aza-NP and free 5-Aza drug were stable under frozen storage (–20 °C), degraded progressively under refrigeration (4 °C), and were highly unstable at elevated temperature (30 °C). Importantly, encapsulation of 5-Aza within NPs conferred markedly enhanced stability compared with free 5-Aza under stress conditions, with the most pronounced protective effect observed at 30 °C.

To comprehensively describe the degradation behavior of 5-Aza under different storage conditions, four kinetic models including zero-order, first-order, Higuchi, and Korsmeyer–Peppas were evaluated for both free 5-Aza and 5-Aza-NP (Figure S3). Across all temperatures, the degradation of free 5-Aza and 5-Aza-NP showed the best agreement with an apparent first-order kinetic profile, reflected by high determination coefficients. For free 5-Aza in PBS, first-order fitting yielded R^2^ values of 0.88 at −20 °C, 0.86 at 4 °C, and 0.91 at 30 °C, respectively. Likewise, 5-Aza-NP also followed first-order behavior, particularly at −20 °C (R^2^ = 0.94), 4 °C (R^2^ = 0.81), and 30 °C (R^2^ = 0.81). These model fits were superior or comparable to zero-order kinetics, which exhibited slightly lower R^2^ values for PBS (0.84–0.95) and NP formulations (0.79–0.93), indicating that degradation proceeds proportionally to remaining drug concentration rather than time-dependent uniform loss.

While first-order kinetics provided the primary description of 5-Aza degradation, diffusion-controlled behavior, as modeled by Higuchi kinetics, also showed strong correlations, particularly at sub-ambient temperatures. Higuchi fitting for 5-Aza-NP returned with R^2^ = 0.93 at −20 °C, 0.94 at 4 °C, and 0.94 at 30 °C, whereas free 5-Aza in PBS showed similarly high agreement (R^2^ = 0.93, 0.93, and 0.98, respectively). These high R^2^ values indicate that diffusion-controlled release/degradation processes likely contribute to stability loss, particularly during prolonged refrigerated storage. The Korsmeyer–Peppas model further supported this interpretation, with moderate to strong fits for 5-Aza-NP (R^2^ = 0.80–0.98) and PBS solutions (R^2^ = 0.65–0.90). Taken together, these findings indicate that although first-order kinetics dominate the overall behavior, matrix-like diffusion mechanisms play a non-negligible role, particularly for NP-encapsulated 5-Aza.

First-order kinetic calculations showed that at −20 °C, 5-Aza-NP displayed a half-life and shelf-life of 866.2 h (36.1 days) and 131.7 h (5.5 days). Under the same conditions, free 5-Aza in PBS exhibited a shorter half-life of 486.8 h (20.3 days) and a shelf-life of 74.0 h (3.1 days) (Table 1 and Table 2). At 4 °C, the NP formulation exhibited half-lives of 533.1 h (22.2 days), and shelf-lives of 81.0 h (3.4 days), respectively. In comparison, free 5-Aza showed a half-life of 261.4 h (10.9 days) and a shelf-life of 39.7 h (1.7 days) (Table 2). Under accelerated degradation at 30 °C, both systems showed rapid decay, with t½ of 59.2 h (NP) and t_90_ of 9.0 h, whereas free 5-Aza degraded more rapidly with a half-life of 21.0 h (0.9 day) and a shelf-life of 3.2 h.

**Figure 7:**
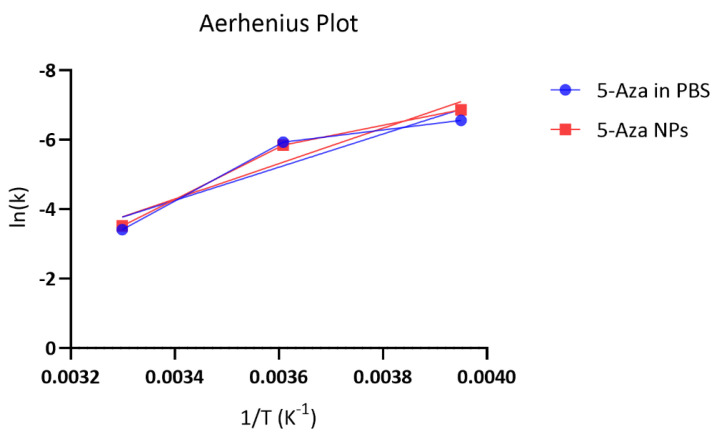
Arrhenius plot of 5-Aza degradation in phosphate-buffered saline (PBS) and in 5-Aza-NPs.

To further characterize the temperature dependence of 5-Aza degradation, Arrhenius plots were constructed using the first-order rate constants obtained at −20, 4, and 30 °C (Figure 7). The linear relationships between lnk and 1/T (K^−1^) yielded good correlations for both free 5-Aza drug and 5-Aza-NP formulations, with coefficients of determination (R^2^) of 0.873 and 0.938, respectively. For the free 5-Aza drug in PBS, the Arrhenius fit was described by the equation lnk = 11.97 − 4772.7(1/T), corresponding to an apparent activation energy (Ea) of 39.7 kJ·mol^−1^ and a pre-exponential factor (A) of 1.58 × 10^5^ h^−1^. In comparison, the 5-Aza-NP-loaded formulation followed the equation lnk = 13.05 − 5102(1/T), with a slightly higher activation energy of 42.4 kJ·mol^−1^ and a pre-exponential factor of 4.6 × 10^5^ h^−1^.

These results demonstrate that degradation of 5-Aza in both formulations is an activated process, with similar energy barriers. The slightly higher Ea observed for the 5-Aza-NP formulation suggests that encapsulation marginally increases the energy required for degradation, consistent with the improved stability observed under frozen conditions. However, the Arrhenius parameters also confirm that at elevated temperatures, the rate constants increase sharply for both free and NP formulations, explaining the limited shelf-lives observed at 30 °C.

### 3.3. DNMT1 Gene Expression in Human Cardiac Fibroblasts Using RT-qPCR

**Figure 8:**
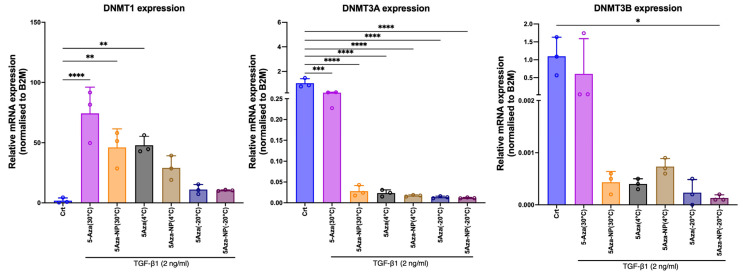
Assessment of *DNMTs* gene expression in human cardiac fibroblasts using RT-qPCR through 5-Aza or 5-Aza-NP stored under various temperature conditions. Data are presented as mean ± SD (n = 3). Asterisks indicate significant differences compared with the corresponding Control: * *p* < 0.05, ** *p* < 0.01, *** *p* < 0.001, **** *p* < 0.0001.

To determine whether the improved physicochemical stability of 5-Aza-NP translated into preserved epigenetic activity, *DNMT* mRNA expressions were quantified in TGF-β1-stimulated HCFs treated with free 5-Aza or 5-Aza-NP that had been stored for 96 h at different temperatures (Figure 8). As expected, *DNMT1* expression was very low in the control group and markedly increased after TGF-β1 stimulation in the presence of 5-Aza stored at 30 °C, whereas 5-Aza-NP (30 °C) and all 4 °C and −20 °C conditions showed progressively lower DNMT1 levels, with the greatest suppression observed for formulations stored at −20 °C. Both free 5-Aza and 5-Aza-NP at 30 °C did not suppress *DNMT1* expression in HCFs treated with TGF-β1, indicating loss of biological activity due to thermal degradation. In contrast, at 4 °C, 5-Aza-NPs markedly suppressed *DNMT1* expression, whereas free 5-Aza stored under the same conditions failed to suppress *DNMT1* expression. Although *DNMT1* expression levels were consistently lower in the 5-Aza-NP group compared with free 5-Aza across storage temperatures, this difference did not reach statistical significance in direct pairwise comparisons.

*DNMT3A* and *DNMT3B* were expressed at much lower absolute levels than *DNMT1* and showed only modest changes across treatment groups. These data indicate that *DNMT1* is the primary epigenetic target affected by 5-Aza in this model and the most sensitive readout for detecting loss or preservation of biological activity after storage.

## 4. Discussion

5-Aza is a clinically approved DNMT inhibitor used for the treatment of myelodysplastic neoplasms and is frequently administered off-label for malignancies such as acute myeloid leukemia [15]. In addition to oncology, epigenetic dysregulation has increasingly been implicated in cardiovascular pathogenesis, with accumulating evidence demonstrating the role of DNA methylation in cardiac remodeling [1]. This has positioned epigenetic modulators, including 5-Aza, as promising candidates for preventing or reversing pathological remodeling [1,16]. However, despite its therapeutic advantages, the clinical utility of 5-Aza remains limited by its poor chemical stability. Our findings show that encapsulating 5-Aza in the NP offers meaningful improvements in both physicochemical stability and functional epigenetic activity.

We observed that storage temperature strongly influenced NP stability. At −20 °C, both blank and 5-Aza-loaded NPs exhibited marked increases in particle size, with blank NPs showing greater aggregation. This behavior is consistent with the known freeze–thaw stress experienced by colloids, where ice crystal formation concentrates particles, disrupts stabilizing interactions, and promotes irreversible aggregation in the absence of cryoprotectants [17]. The instability was further supported by the progressive increase in PDI values, indicating broader particle size distributions and reduced homogeneity.

In contrast, NP formulations stored at 4 °C demonstrated the greatest stability, with consistently low PDI values and moderately negative surface charges. These results indicate that refrigerated storage is optimal for maintaining dispersion quality and agree with prior studies showing that reduced temperatures minimize particle kinetic energy and help prevent aggregation in nanosystems [18,19]. Storage at 30 °C yielded acceptable size and PDI values, and the more negative zeta potentials observed may have enhanced electrostatic stabilization.

At 4 °C, the zeta potential of 5-Aza-NP shifted toward less negative (i.e., more positive) values compared with blank NPs (Figure 4B), indicating a reduction in the magnitude of the negative surface charge. This shift is consistent with partial charge neutralization of the hyaluronic acid (HA) coating on the nanoparticle surface following drug loading. According to DrugBank (ID: DB00928), 5-Aza exhibits pKa values of approximately 1.96 and 12.55, suggesting that under formulation conditions, the molecule exists predominantly retaining localized partial positive character on its heterocyclic nitrogen atoms. These protonated sites can interact electrostatically with negatively charged carboxylate groups of HA, thereby partially neutralizing the surface charge and shifting the zeta potential toward more positive values.

In contrast to the behavior observed at −20 and 4 °C, the zeta potential of 5-Aza-loaded NPs became more negative at 30 °C over time. This trend may be attributed to the accelerated degradation of 5-Aza at elevated temperature, which reduces the availability of intact drug molecules capable of interacting with and partially neutralizing the negatively charged HA surface. As the concentration of surface-associated 5-Aza decreases due to degradation, the intrinsic negative charge of the HA coating becomes increasingly exposed, resulting in a more negative apparent zeta potential.

The chemical instability of 5-Aza is well established and stems from the rapid hydrolysis of its cytidine analog structure. In aqueous environments, triazine ring opening and subsequent hydrolysis produce inactive degradation products, a process intensified by neutral-to-alkaline pH and higher temperatures [20]. Additional pathways, including deamination and structural rearrangements, further accelerate drug degradation [21,22,23,24]. NP encapsulation provided partial protection against these processes. Under frozen storage, the NP matrix likely reduced drug exposure to water through restricted molecular mobility and lower free energy, thereby slowing degradation [25]. At 4 °C, however, sufficient water penetration into the polymeric matrix allowed hydrolysis to continue, demonstrating that encapsulation alone cannot fully prevent degradation at this temperature. At 30 °C, NPs modestly slowed late-phase degradation, but this effect was insufficient to counteract the dominant influence of temperature-driven hydrolysis. Considering both stability and practical handling, storage at 4 °C is recommended for short-term use, as it avoids freeze–thaw stress while maintaining acceptable stability; long-term preservation still necessitates frozen storage at −20 °C or lower.

The temperature-dependent instability observed in our system aligns well with previous reports demonstrating that 5-Aza is highly sensitive to thermal conditions. Walker et al. showed that reconstituted 5-Aza undergoes rapid degradation at room temperature, losing approximately 15% of the initial concentration after 9.6 h; at 4 °C, 32% was lost after 4 days; and at −20 °C, less than 5% was lost after 23 days. These results reinforce our kinetic predictions, which showed a dramatic reduction in half-life when storage temperature increased from frozen to ambient conditions [7].

To assess whether NP encapsulation preserves the biological function of 5-Aza, *DNMT* expression profiles were evaluated following TGF-β1 stimulation in HCFs treated with free or NP-loaded 5-Aza stored under various temperatures for 96 h. As expected, *DNMT1* expression was minimal in untreated cells and markedly increased after TGF-β1 exposure. Free 5-Aza stored at 30 °C displayed a substantial loss of inhibitory activity, whereas 5-Aza–NPs stored at 30 °C showed similar reductions, indicating that NP encapsulation cannot protect the drug at elevated temperature. In contrast, at 4 °C, the difference between formulations became evident: free 5-Aza showed only partial *DNMT1* suppression, while 5-Aza–NPs maintained expression levels near baseline, demonstrating superior preservation of *DNMT1*-inhibitory activity. At −20 °C, both free and NP-encapsulated 5-Aza retained strong *DNMT1* inhibition, reflecting the minimal degradation that occurs under frozen conditions. The differences observed among the 5-Aza-NP groups were not attributable to variability in the carrier itself but rather reflected temperature-dependent preservation of 5-Aza bioactivity within the nanoparticle matrix. Consistent *DNMT1* suppression in stored 5-Aza-NP samples supports a stabilizing role of the formulation rather than an independent effect of the carrier on gene expression.

Unlike *DNMT1*, *DNMT3A* and *DNMT3B* expression remained consistently low and did not show clear induction across treatment conditions compared with controls, regardless of formulation or storage temperature. This stability reflects their biological roles as de novo methyltransferases, which are expressed at low levels in HCFs and are less responsive to cytidine analog-based inhibition [25]. Consequently, *DNMT3A* and *DNMT3B* do not provide sensitive indicators of 5-Aza functional degradation in this model. The higher *DNMT3A* and *DNMT3B* expression observed in HCFs + TGF-β1 treated with free 5-Aza stored at 30 °C, compared with free 5-Aza stored at 4 °C and −20 °C, may be attributable to thermal degradation of the drug, resulting in reduced DNMT inhibitory activity. In this context, sub-therapeutic exposure may be insufficient to induce effective DNMT trapping and could instead elicit a compensatory response, including *DNMT3B* upregulation associated with incomplete *DNMT1* inhibition, potentially diminishing the efficacy of 5-Aza [26]. Collectively, these findings suggest that free 5-Aza is temperature sensitive and highlight the importance of appropriate storage conditions for maintaining its biological activity, with *DNMT1* emerging as a reliable biomarker for evaluating the preservation of 5-Aza activity after storage.

In summary, NP encapsulation substantially improves the physicochemical stability and biological function of 5-Aza, extending its usable lifespan beyond that of the reconstituted drug alone. Although the intrinsic chemical fragility of 5-Aza remains the major limitation, this study identifies a viable formulation strategy that enhances stability under clinically relevant temperature conditions. These results highlight the potential of NP-based delivery systems to stabilize labile epigenetic drugs and broaden their therapeutic applicability.

## Figures and Tables

**Table 1:** Comparison of first-order half-life (t½) of free 5-Aza in PBS and 5-Aza-NP.

Temperature	t½ (h) 5-Aza (PBS)	t½ (Days) 5-Aza (PBS)	t½ (h) 5-Aza-NP	t½ (Days) 5-Aza-NP	Stability Gain (NP vs. Free Drug)
−20 °C	486.8	20.3	866.2	36.1	~1.8-fold
4 °C	261.4	10.9	533.1	22.2	~2.0-fold
30 °C	21.0	0.9	59.2	2.5	~2.8-fold

**Table 2:** Comparison of first-order shelf-life (t_90_) of free 5-Aza in PBS and 5-Aza-NP.

Temperature	t½ (h) 5-Aza (PBS)	t½ (Days) 5-Aza (PBS)	t½ (h) 5-Aza-NP	t½ (Days) 5-Aza-NP	Stability Gain (NP vs. Free Drug)
−20 °C	74.0	3.1	131.7	5.5	~1.8-fold
4 °C	39.7	1.7	81.0	3.4	~2.0-fold
30 °C	3.2	0.13	9.0	0.38	~2.8-fold

## Data Availability

The original contributions presented in this study are included in the article/Supplementary Materials. Further inquiries can be directed to the corresponding author.

## Supplementary Materials

The following supporting information can be downloaded at: https://www.mdpi.com/article/10.3390/pharmaceutics18010088/s1, Figure S1. Number-based dynamic light scattering (DLS) size distribution of (A) 5-AZA-NP and (B) Blank-NP. Figure S2. Representative HPLC chromatograms of 5-Aza standard, 5-Aza in PBS, and 5-Aza-loaded nanoparticles (5-Aza-NP). Individual chromatograms of the 5-Aza standard (green), 5-Aza in PBS (blue), and 5-Aza-NP (red) are shown together with an overlay plot for direct comparison. Figure S3. Kinetic modeling of 5-Aza-NP (red symbols and lines) and 5-Aza in PBS (blue symbols and lines) degradation profiles using zero-order, first-order, Higuchi, and Korsmeyer–Peppas equations at different storage temperatures (−20, 4, and 30 °C). (A,E,I) correspond to zero-order plots (drug remaining versus time); (B,F,J) depict first-order models (log drug remaining versus time); (C,G,K) show Higuchi diffusion plots (drug remaining versus square-root of time); and (D,H,L) illustrate Korsmeyer–Peppas behavior (log drug remaining versus log time).
