# Supplementary material for: A Nanoparticle-Based Strategy to Stabilize 5-Azacytidine and Preserve DNA Demethylation Activity in Human Cardiac Fibroblasts
Source: Pharmaceutics. 2026 Jan 9;18(1):88. doi: 10.3390/pharmaceutics18010088 (PMC12845215; doi:10.3390/pharmaceutics18010088)
Supplement: Supplementary file 1 [file pharmaceutics-18-00088-s001.zip › pharmaceutics-4050693-supplementary.pdf]

A

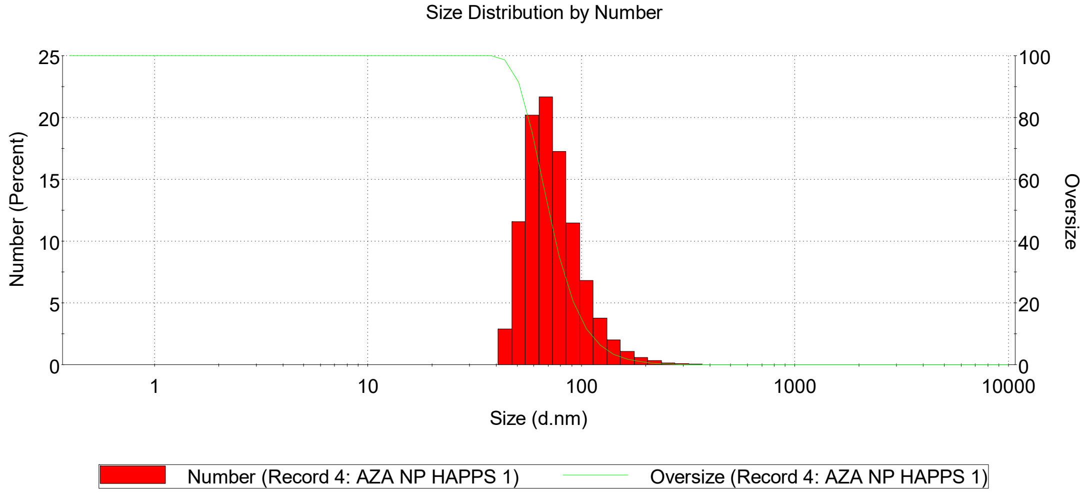

B

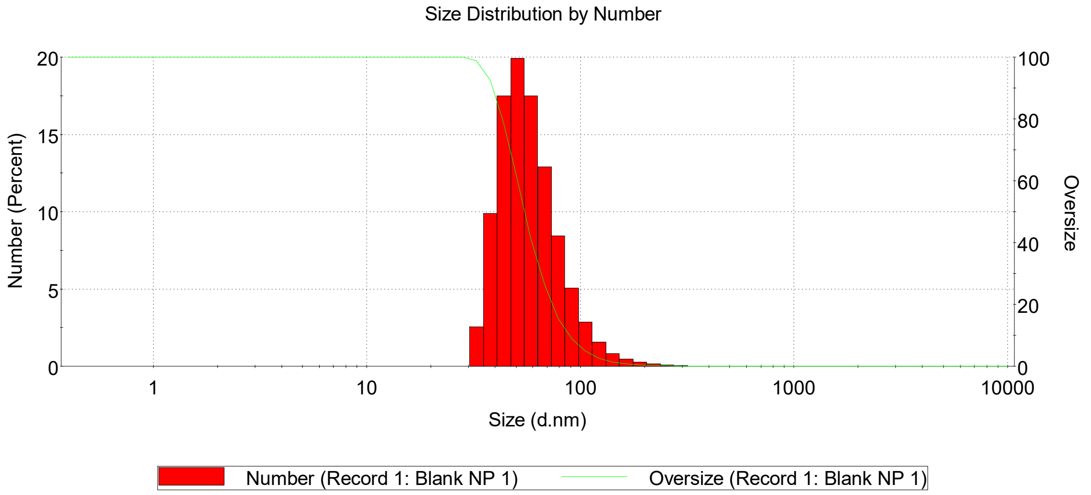

Figure S1. Number-based dynamic light scattering (DLS) size distribution of (A) 5-AZA-NP and (B) Blank-NP

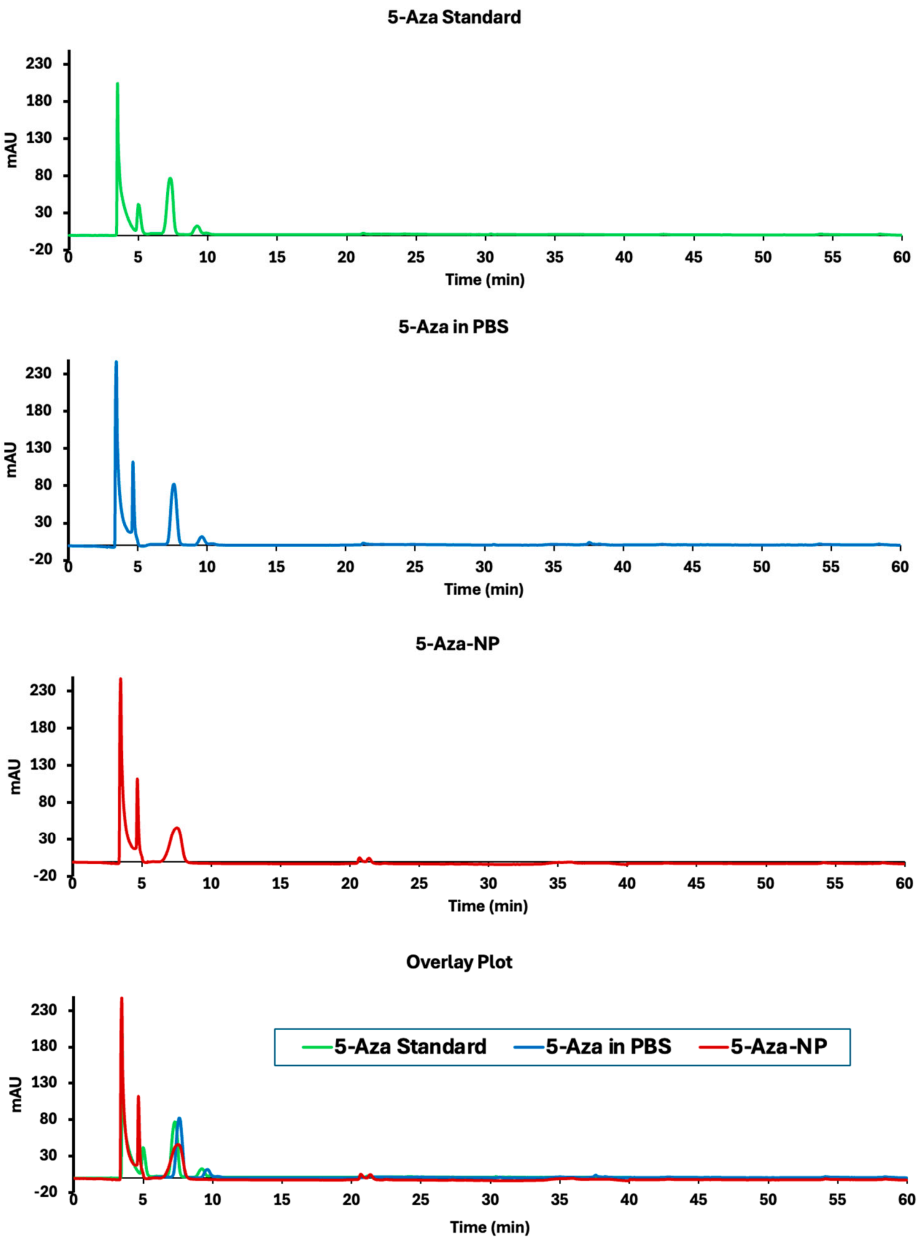

Figure S2. Representative HPLC chromatograms of 5-Aza standard**,** 5-Aza in PBS, and 5-Aza-loaded nanoparticles (5-Aza-NP)**.** Individual chromatograms of the 5-Aza standard (green), 5-Aza in PBS (blue), and 5-Aza-NP (red) are shown together with an overlay plot for direct comparison.

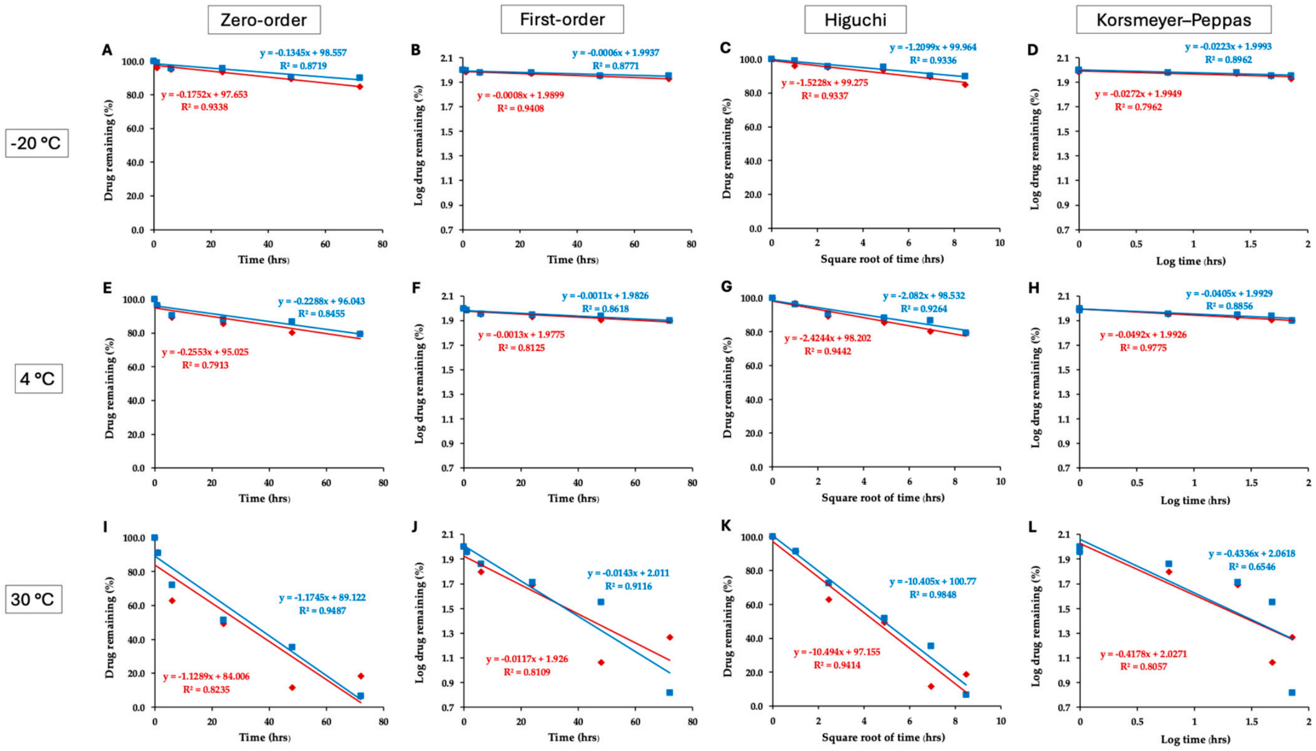

Figure S3. Kinetic modeling of 5-Aza-NP (red symbols and lines) and 5-Aza in PBS (blue symbols and lines) degradation profiles using zero-order, first-order, Higuchi, and Korsmeyer–Peppas equations at different storage temperatures (-20, 4, and 30 °C). A, E, and I correspond to zero-order plots (drug remaining versus time); B, F, and J depict first-order models (log drug remaining versus time); C, G, and K show Higuchi diffusion plots (drug remaining versus square-root of time); and D, H, and L illustrate Korsmeyer–Peppas behavior (log drug remaining versus log time).
